# Light-Weight Integration and Interoperation of Localization Systems in IoT

**DOI:** 10.3390/s18072142

**Published:** 2018-07-03

**Authors:** Abdulkadir Karaagac, Pieter Suanet, Wout Joseph, Ingrid Moerman, Jeroen Hoebeke

**Affiliations:** 1 Department of Information Technology, Ghent University/imec, IDLab, Technologiepark-Zwijnaarde 15, B-9052 Ghent, Belgium; wout.joseph@ugent.be (W.J.); ingrid.moerman@ugent.be (I.M.); jeroen.hoebeke@ugent.be (J.H.); 2 Aucxis CVBA, Zavelstraat 40, 9190 Stekene, Belgium; pieter.suanet@aucxis.com

**Keywords:** localization, Internet of Things (IoT), positioning, semantic interoperability, machine to machine (M2M), Lightweight M2M (LwM2M), Internet Protocol for Smart Objects (IPSO)

## Abstract

As the ideas and technologies behind the Internet of Things (IoT) take root, a vast array of new possibilities and applications is emerging with the significantly increased number of devices connected to the Internet. Moreover, we are also witnessing the fast emergence of location-based services with an abundant number of localization technologies and solutions with varying capabilities and limitations. We believe that, at this moment in time, the successful integration of these two diverse technologies is mutually beneficial and even essential for both fields. IoT is one of the major fields that can benefit from localization services, and so, the integration of localization systems in the IoT ecosystem would enable numerous new IoT applications. Further, the use of standardized IoT architectures, interaction and information models will permit multiple localization systems to communicate and interoperate with each other in order to obtain better context information and resolve positioning errors or conflicts. Therefore, in this work, we investigate the semantic interoperation and integration of positioning systems in order to obtain the full potential of the localization ecosystem in the context of IoT. Additionally, we also validate the proposed design by means of an industrial case study, which targets fully-automated warehouses utilizing location-aware and interconnected IoT products and systems.

## 1. Introduction

Over the past few years, we have been experiencing a fast emergence of the Internet of Things (IoT), where things and objects become smart and connected [1]. By providing access to and interaction with a broad range of devices and systems, the IoT is fostering the development of numerous applications and services in many different domains, such as industry, building automation, smart grids, smart cities, healthcare, wearables and many others [2]. In many cases, these applications can greatly benefit from or cannot properly function without location awareness and the ability to identify the location of objects (sensors, machines, wearable devices).

Besides IoT, indoor localization is another growing trend in our hyper-connected society with a large amount of research and industrial focus [3]. Moreover, the demand for Location-Based Services (LBSs) has been rapidly expanding in many fields, such as goods/robot tracking in industry or indoor navigation for people with visual impairments, and is supported by the emergence of several powerful commercially-available localization solutions [4]. Due to their diverse positioning techniques and technologies, each localization solution has unique capabilities, limitations and also varying costs. For instance, although GPS is the de-facto positioning solution for outdoor environments, Ultra-Wideband (UWB) or LoRa-based technologies can be the first choice for applications that only have high positioning accuracy or low accuracy, but long-range and low-energy requirements, respectively [4,5,6]. Despite this plethora of technologies and the remarkable interest in the localization domain, the interoperation and orchestration of these technologies and their integration into other ecosystems is still an open issue that requires innovation.

In this sense, we see several standardization initiatives and research efforts targeting interoperability and Machine-to-Machine (M2M) understandability in the IoT domain. These efforts aim to create more loosely-coupled systems, better interoperable and connective devices via common interfaces and data models. For instance, oneM2M [7], the Open Mobile Alliance (OMA) [8] and the Internet Protocol for Smart Objects (IPSO) Alliance [9] are some of the leading global organizations that deliver specifications and architectures for creating resource-efficient M2M communication and global interoperability for the IoT. However, there is no effort available that extensively targets the interoperability of localization technologies by considering their specific characteristics or constraints.

In this work, we study the first design for the interoperation and integration of positioning systems by means of IoT application protocols and data models, more specifically, the ones defined by OMA Lightweight Machine-to-Machine (LwM2M) [10] and IPSO. For this purpose, we create a uniform and well-defined representation for the location semantics combined with interaction models that can be used for dealing with various localization technologies and system interactions. We show that these IoT protocols and specifications, by design, offer the necessary mechanisms and features in order to obtain syntactic and semantic interoperability between positioning systems and also IoT applications. In addition, we also validate our approach by means of an industrial use case and system implementation, which targets fully-automated warehouses by means of location-aware and interconnected IoT products and systems.

We believe that such an integration will enable a seamless and spontaneous interoperation of localization technologies independent of their specific characteristics, hardware and software components. This will also enable IoT applications to simultaneously use a multitude of diverse positioning solutions in order to face various application needs or improve overall localization performance.

The remainder of this paper is organized as follows. Section 2 provides detailed background on the localization technologies and the LwM2M protocol in order to understand the current state of the art. The vision and the necessary enablers for the integration of IoT and localization technologies are presented in Section 3. The mechanisms that constitute the fundamentals of the light-weight integration and interoperation of the localization technologies are described in Section 4. Section 5 describes how the proposed solution can be exploited in order to achieve interoperability for different interaction models in the localization ecosystem, which is followed by a case study, in Section 6, that validates the proposed solution and illustrates the main principles and application potential. Finally, Section 7 concludes the paper.

## 2. Background

### 2.1. Localization Systems: The World of Diversity

Localization is the process of determining the position of equipment, people or any other object (called the tag in this paper). In the past decade, it has become an active research area. Up to now, a large variety of localization technologies have been proposed, but no one-size-fits-all solution has emerged [3]. Therefore, today, we face an enormous and heterogeneous ecosystem with varying localization technologies, techniques, architectures and designs. Although there are various ways to classify these technologies, this section will only provide their key aspects that are relevant to the focus of this paper.

First of all, there is a wide variety of technologies on which localization systems can be based, such as WiFi, BLE, RFID, UWB, LoRa, GPS, ultrasound and vision [3]. Some of these technologies are already available on several commercial devices, whereas some of them are composed of extremely specialized and expensive components. Secondly, the environment in which the localization is conducted is one of the most important aspects of these systems. Although there are two types of solutions (indoor vs. outdoor), even the characteristics of the indoor environment play a key role in the performance of the localization technologies [4]. Thirdly, localization systems use various measurement types and positioning techniques in order to calculate the position of the target object. Independent of their technology, Time of Arrival (ToA), Angle of Arrival (AoA), Time Difference of Arrival (TDoA) and Received Signal Strength (RSS) are the most commonly-used techniques applied by localization technologies. Finally, we also see variation in the type of system architecture. Some of the technologies use a self-positioning architecture, in which objects calculate their position themselves. There are also systems that include an infrastructure or backend server that calculates the location of the targets. Finally, we see self-oriented infrastructure-assisted architectures where a backend system determines the position and then informs the tracked object in response to its request [4].

On the other side, different applications may also ask for different localization requirements in terms of various quality metrics, such as accuracy, precision, scalability, update frequency, etc. [5]. This diversity of technologies and the variation in the application requirements prevents the emergence of a single solution that is a silver bullet for creating a localization solution that meets all possible needs. Therefore, for each application, a suitable localization technology should be selected wisely in order to meet application needs while maintaining the right balance between the system cost, complexity and performance. Often, the most efficient solution is a combination of technologies and techniques especially for complex applications with various needs.

Regarding the interoperation of localization systems, we recently have started to see a number of research efforts that are investigating semantic location models, mathematical methods, ontologies and structures for location-based applications with no resource constraints, especially related to location navigation and browsing [11,12,13,14]. However, in pursuit of the interoperation of this large variety of localization systems, one needs to consider flexible and powerful mechanisms that consider all aspects and variations of the localization technologies.

### 2.2. Lightweight Machine-to-Machine Protocol

**Figure 1:**
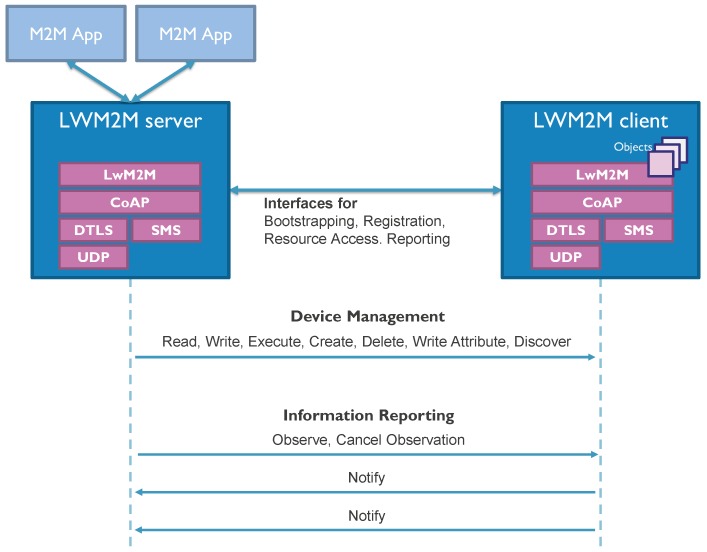
Open Mobile Alliance (OMA) LwM2M interaction model.

LwM2M, specified by the OMA Alliance, is a secure, efficient and deployable client-server protocol with several functionalities for managing resource-constrained devices on a variety of networks. Besides fundamental management functionalities such as bootstrapping, client registration and firmware updates, LwM2M also defines efficient interactions for remote application management and the transfer of service and application data [10]. For this purpose, LwM2M provides several interfaces built on top of the Constrained Application Protocol (CoAP) [15], which is a REST-based application protocol for constrained Internet devices. Figure 1 presents the LwM2M interaction model related to device management and information reporting. As it makes use of light and compact application protocols, management mechanisms and an efficient resource data model, the LwM2M protocol has already attracted much attention from the research community.

According to LwM2M, a client consists of one or more instances of objects, which are typed containers that define the semantic type of instances. Each object is a collection of mandatory and optional resources, which are atomic pieces of information that can be read, written or executed. These objects, instances and resources are mapped into the URI path hierarchy with integer identifiers and can be accessed via simple URIs in the form of /ObjectID/InstanceID/ResourceID [10]. For instance, a device model number can be read via a GET request to the URI “/3/0/1”. At the time of writing, there are more than 100 objects registered, by OMA Working Groups, third party organizations, vendors and individuals, via a registration process with the OMA Naming Authority (OMNA) [16]. Among these object types, there are two location-related objects: location object from OMA and GPS location from IPSO. However, since both of these objects can only be used to represent GPS location data, these models are not sufficient for use by all localization systems, especially for non-spatial data (*X*,*Y*,*Z*).

Next to OMA, the IPSO alliance performs a similar and adherent effort in order to provide a common design pattern and an object model based on the OMA LwM2M specification [9]. By using reusable object and resource design, IPSO targets high level interoperability between smart object devices and connected software applications on other devices and services [17].

Thanks to the use of open IoT standards, unified information and interaction models and powerful management functions, LwM2M is a very promising candidate to achieve global interoperability within the IoT Ecosystem, especially when constrained devices are involved. Therefore, within the IoT ecosystem, there are ongoing efforts defining mechanisms and protocols in order to realize semantic and structural interoperability. However, there is no effort available that extensively targets the interoperability of localization technologies by considering their specific characteristics or constraints. Therefore, in this work, we tried to leverage the LwM2M protocol to target interoperability and integration of localization systems in IoT.

## 3. IoT Interoperability for Localization Systems

**Figure 2:**
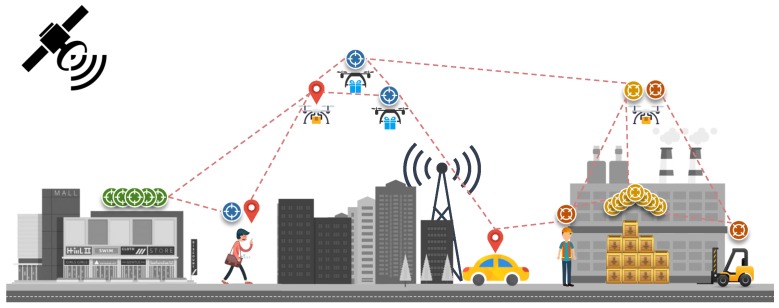
The vision for the integration of the localization and IoT technologies.

The successful integration of IoT and localization technologies is mutually beneficial and even essential for both fields. Such an advancement would enable numerous new IoT applications and products with location awareness and location-based reasoning. On the other side, multiple localization systems would communicate and interoperate with each other in order to obtain better context information, improve localization accuracy, resolve positioning errors or conflicts and activate/inactivate each other in order to save resources in various conditions. Ultimately, all these new applications and features will result in a broader and smarter ecosystem, as illustrated in Figure 2, with significant potential: smarter “smart cities”, “smart factories”, “smart buildings”, etc.

However, despite the plethora of technologies and the remarkable progress made in these domains, their integration, interoperation and harmonization is still an open issue. Considering the diversity of localization technologies, as described in Section 2.1, and the scalability and efficiency concerns in IoT, the success of such an integration becomes a very hard mission, and it can only become a reality with the design of flexible information and interaction models and powerful and efficient management functionalities.

**Figure 3:**
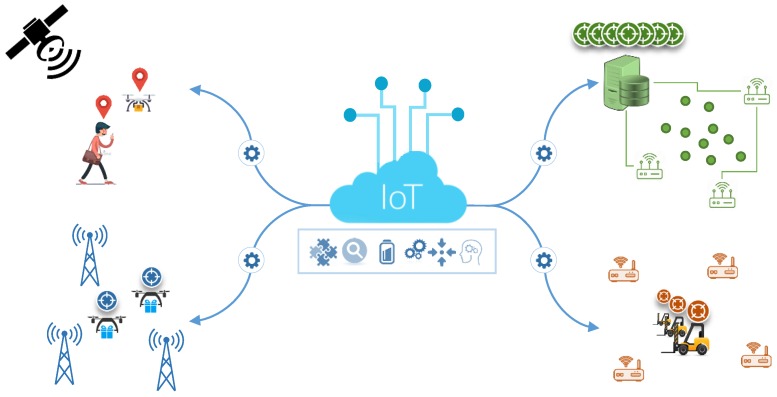
The integration of localization technologies.

In this context, we defined the following functionalities as vital enablers for the realization of full structural, syntactical and semantic interoperability between localization systems and also IoT applications, as illustrated in Figure 3, with minimized integration cost.

First of all, the concerns about the security and privacy of localization data must be sufficiently addressed, so only authenticated and authorized parties can reach location data and perform trustworthy operations in a security domain. Especially, considering large-scale IoT scenarios, a vast number of localization devices and systems needs to be authenticated and authorized along with a wide range of smart objects.

Secondly, the vision of the seamless interconnection of localization technologies necessitates mechanisms for automatic discovery of devices, resources, their properties and capabilities, as well as the means to access them. Therefore, any application or device can discover these devices (localization tags, systems) with positioning capabilities, along with their types and settings. Furthermore, such discovery mechanisms also depend on other services like configuration management, registration and un-registration of self-descriptive devices, systems and resources.

Moreover, considering that the majority of IoT devices are expected to be severely constrained in terms of memory, CPU and power capacities, the interoperability solution must embody a light, compact, efficient and scalable nature. Therefore, it can easily adapt to constrained environments and large-scale deployments (support for tracking of thousands of devices).

Next, there has to be a common dictionary (uniform data models) describing formally-relevant concepts, resources, attributes and relations without ambiguity in order to achieve semantic interoperability and global understandability. This will enable systems to perform machine computable logic, knowledge discovery, data federation and semantic-based reasoning. In addition, along with essential location data, further additional information (reference point, orientation, etc.) has to be reachable for other applications and devices, so interested parties can correlate positions from different localization technologies and map them to other coordinate systems.

Finally, in pursuit of the interoperation of the large variety of localization systems, the interoperability solution needs to consider all aspects and variations of the localization technologies (e.g., spatial vs. non-spatial data) and provide support for all localization architectures (self-positioning, infrastructure-based or infrastructure-assisted localization).

For this purpose, we study a design for the interoperation and integration of positioning systems by means of IoT application protocols and data models defined by OMA LwM2M and IPSO. We show that, with extensions of uniform and well-defined data and interaction models targeting localization systems, these IoT protocols and specifications, by design, offer the necessary mechanisms and features in order to obtain syntactic and semantic interoperability between positioning systems and also IoT applications.

## 4. Light-Weight Integration and Interoperation of Localization Systems in IoT

In this section, we first present the mechanisms in the LwM2M protocol that constitute the fundamentals (defined in the previous section) of the full integration and interoperation of the localization technologies, including bootstrapping, resource registration, operations and common system architectures. Then, we describe the designed uniform object models, based on LwM2M/IPSO specifications, that can be used to inclusively represent the location-related data from various and diverse localization technologies. Finally, for each aspect of the architecture, we describe the overall flow of the interoperation process based on the proposed solution.

### 4.1. Bootstrapping, Registration and Discovery

Bootstrapping is an LwM2M functionality that is used for server configuration, security and credential management, as well as provisioning of access control lists [10]. For client-based bootstrapping, a dedicated LwM2M bootstrap server is used, which is a specific server that is contacted by the client during its boot-up and prepares the client for communication with regular LwM2M servers. Considering the privacy and security concerns regarding the localization technologies [18], the LwM2M bootstrap interface offers key functionalities, by means of credentials and access control management, in order to prevent unauthorized operations on positioning data.

Device registration is another LwM2M feature that allows a LwM2M client device to inform an LwM2M server about its existence and register its capabilities and resources [10]. This way, the LwM2M server can act as a lookup server, enabling any application to perform queries and discover all devices with positioning capabilities.

**Figure 4:**
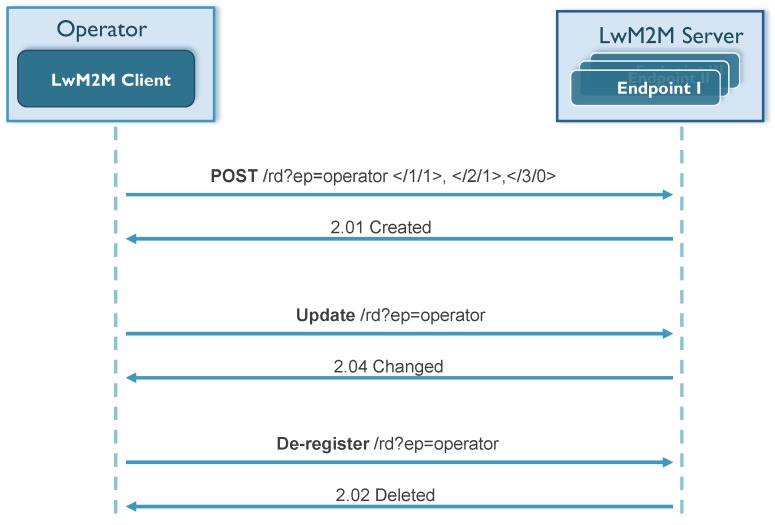
LwM2M resource registration example flow.

This not only allows device registration, but also enables a discovery of the application/device to understand what kind of location-related objects a device holds and which resources are exposed by the particular object. Further, by identifying object IDs and/or reading the descriptive resources (e.g., application type), additional and detailed information can be retrieved. Next, it can start reading the location data, retrieving the information it is interested in and also performing all the operations defined in the following section. In Figure 4, the LwM2M registration process is illustrated with a flow diagram. Besides the registration operation, an LwM2M client can also update its registration or perform a de-registration when shutting down or discontinuing use of an LwM2M server [10].

### 4.2. Light-Weight and Efficient Operations

Since LwM2M relies on the CoAP application protocol, the CoAP methods constitute the fundamentals of the LwM2M interactions and operations. A minimal CoAP request consists of the method to be applied to the resource, the identifier of the resource, a payload and metadata about the request [15]. CoAP supports the basic methods of GET, POST, PUT and DELETE. The CoAP GET method is the fundamental information retrieving method, whereas the PUT method is used to update the resource identified by the requested URI, and the POST method usually results in a new resource being created or the target resource being updated [15]. Besides these basic CoAP methods, there have been recent efforts to define new CoAP methods in order to create CoAP applications with improved functionalities. The newly-specified FETCH, PATCH and iPATCHmethods allow accessing and updating parts of a resource [19]. In addition to the basic methods, these new methods can be very beneficial for dealing with location data. For instance, an application can read *X*, *Y* and *Z* coordinates of a localized object with a single FETCH request, upon which it will only receive the related data aggregated in a single packet. Although the current LwM2M specification does not support CoAP PATCH/FETCH functionality, the preview of the next version of the LwM2M specification declares that these methods will be included in the near future [20].

The LwM2M protocol also defines an information reporting interface (based on the CoAP observe mechanism), which can be used to achieve object tracking by means of the repeating retrieval of the location data [21]. Using this mechanism, a device, called the observer, can indicate that it is interested in observing a location object instance and to be notified about any state change of the relevant data. This way, the device will periodically notify the observer with a single message that contains the value of the observed resource or the set of resource values for the observed object instance aggregated in one packet.

### 4.3. LwM2M Position Object Models

As we mentioned in the previous sections, LwM2M relies on uniform object models, which are collections of mandatory and optional resources representing atomic pieces of information. Therefore, we create powerful location-related objects and resources that can be used to represent spatial and non-spatial location data in various technologies.

In Table 1, the list of location-related object models is provided. The first object, GPS location (3336), is the location object defined by the IPSO alliance to represent GPS localization data, such as latitude, longitude, uncertainty and velocity. The details and defined resources for this object are listed in Table 2. This object provides the model for spatial location data, but it is too limited considering the indoor localization technologies because most of the indoor localization technologies provide a relative position (in coordinates) with respect to a reference point.

Therefore, we define three new object models, namely position object, localization relay object and localization server object. The position object (3360) provides the necessary resources to represent coordinate-based localization data, whereas an instance of the localization relay object (3361) will expose all necessary resources to associate and link the tag with a localization server, which can provide its location data. Finally, the localization server object (3362) can be used to expose the localization server itself as an LwM2M device. The last two object models can be used for infrastructure-based localization systems. The details of these models are provided in Table 3, Table 4 and Table 5, respectively. In these models, the IDs for the proposed/created objects and resources (3360, 3361, 5552, 5553, etc.) are not standardized, but these IDs have been selected during the design as they were not assigned according to the LwM2M object and resource registry [16]. However, a final ID assignment should go via OMA registration procedure. In addition, as multiple instances can be created by client devices, these devices can expose multiple position objects through several instances.

For each resource, the ‘Operations’ field indicates the supported operations by this specific LwM2M resource. As can be seen in Table 3, unlike the IPSO GPS location object, the resources in the position object support both read and write operations. This feature is essential in order to enable localization servers to write the position data to the client device when using infrastructure-assisted localization technologies. By means of the LwM2M bootstrap interface and the security features of LwM2M, only the authorized servers and devices that have the right device management credentials can perform such write operations on the position object resources.

As is presented in Table 3, the position object is composed of several location-related resources, which are mostly defined in the IPSO specification. For this object, ‘*X* value’ and ‘*Y* value’ are mandatory resources and have to be defined in any position object. The ‘*Z* value’ is optional due to the existence of 2D localization systems. The optional minimum and maximum values for the *X*, *Y* and *Z* coordinates can be used to define the measurement area in which localization is performed. ‘Sensor unit’ defines the unit in which the coordinate measurements are expressed. The ‘uncertainty’ can be used to deliver the accuracy of the latest localization measurement, so third parties can evaluate the location data accordingly. ‘Timestamp’ exposes crucial information regarding the age of the location data. As the tracked objects are mobile, location data are valid for a certain amount of time. The timestamp is also useful when someone tries to combine or match location data from two or more different resources in order to improve location accuracy.

The position object also offers the ‘latitude’, ‘longitude’, ‘altitude’, ‘compass direction’ and ‘elevation direction’ resources, which can be used in order to specify the actual position of the reference point and the relative orientation of the measurement area with respect to this reference point. By mapping reference points of the localization systems, any location data can be translated between two systems, which enables the interoperation of multiple localization systems.

‘Server URI’ can be used to link the position object with the corresponding localization server, if existing. Then, the client application can retrieve detailed information about the localization technology and system setup, such as the number of anchors, the number of tracked objects and any supported feature (maximum update rate, accuracy, etc.). The ‘target ID’ resource can be used to read the unique ID exposed by the tag or assigned by the localization server, while the ‘update flag’ is used to notify that the position data have been last updated by a localization server. Finally, ‘application type’ defines a string resource where system developers can embed any kind of information regarding the localization system. For instance, “cm-level UWB localization technology” would show that this application is exposing really accurate location data for tracked objects.

The localization relay object (3361) is an object we created in order to expose the necessary resources to associate and link the target tag with a localization server. This object is necessary for the localization systems where the location is calculated by a backend server. This way, the tag can relay any request to the corresponding backend server by means of created ‘server URI’ and/or ‘tag ID’ resources. The ‘server URI’ resource can provide an URI (in string format) that identifies the backend server itself or any instance or resource available on the server. Any client first needs to retrieve the server URI and/or the tag ID, and then, it can start retrieving the location data directly from the localization server.

The localization server object (3362) is another proposed object model used to represent a single localization server, which tracks several objects, but only exposes proprietary API. Apart from the ‘application type’, this object exposes only a ‘location server’ resource, which can be used to describe a proprietary API via a string written in JSON format. This server object could also encompass technology-related resources (5508 to 5515, 5552, 5701, etc.) in order to expose more information about the localization system.

## 5. The Realization of Interaction Models for Localization Systems

As is mentioned in the Background section, the architecture of localization systems is one of the features that the localization technologies divert. There are technologies that use a self-positioning architecture, in which objects calculate their positions by themselves. Besides GPS, which is the best-known self-positioning technology, there are also several indoor localization technologies where the tag positions itself by interacting with fixed anchor devices that have known positions, such as the Pozyx UWB Accurate Positioning System [22]. On the other hand, there are also systems that include an infrastructure or backend server that calculates the location of the targets. Most of the TDoA- and AoA-based localization technologies apply this architecture, such as Quuppa’s BLE-based positioning system [23]. Finally, we also see self-oriented infrastructure-assisted architectures where a backend system determines the position and then informs the tracked object. In the following subsections, we describe how our LwM2M–based approach can incorporate any of these localization system architectures.

### 5.1. Self-Positioning

In this interaction model, the target device is calculating its own position, and it can directly expose any measured data via the proposed position object model (3360) in case the technology is not GPS. Any device interested in this information can directly interact with the tag device and read any relevant location information after the resource discovery. If the device exposes multiple location data with different technologies, then the application can reach the data via different position instances (*3360/0, 3360/1, 3360/2*, etc.), and it can also read the ‘application type’ or ‘server URI’ resources to find out the corresponding localization system. In order to track target objects, the application server can send an observe request to the target tag and receive notifications whenever the tag calculates a new position. Besides the data delivery and monitoring, the LwM2M device management interfaces can also be used to configure the tags with the necessary information (e.g., position of anchors) in order to calculate their own positions. Again, with uniform data models, such an interface can offer a generic solution for the tag configuration; however, this is not in the scope of this paper.

### 5.2. Self-Oriented Infrastructure-Assisted

There are also applications where the tag needs the location information, but the location data are measured at the localization system infrastructure. In this case, the authorized localization server needs to determine the position and then inform the tracked object. In such systems, the localization server first discovers the tracked object with the unique ‘tag ID’ and retrieves its IP address. Then, whenever a new location is detected, the localization server (with access rights) can write to the particular position object resources exposed by the tag and set the ‘update flag’. Our position object model enables this operation by exposing all resources as both writable and readable. At the same time, a third party application can read or observe these resources on the tag whenever the server updates the location data.

### 5.3. Infrastructure Backend System

For infrastructure-backend localization systems, there can be four approaches for using our LwM2M object and resource model in order to expose location data.

**Figure 5:**
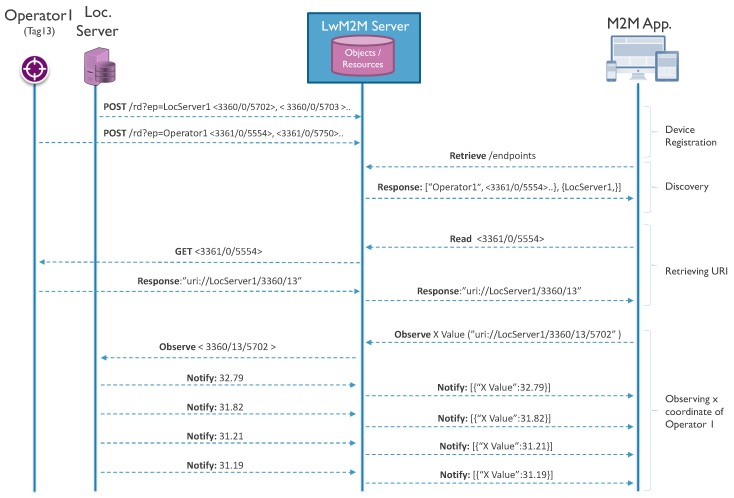
Infrastructure backend one-to-one interaction model: example flow.

Using the first approach, the infrastructure server exposes position data for every tag that is being localized via a dedicated instance of a position object. The link between this object and the tag is created by including the ‘target ID’ resource in this position object instance, with the ID being unique for each tag. At the other side, each tag exposes a localization relay object that contains the ‘server URI’ resource, which constitutes a one-to-one link to the position object instance at the server side. During the bootstrapping process, the infrastructure server needs to create and assign position object instances for the new target tags and inform each tag about the address and URI of the specific position object instance. Whenever an application server would like to read the location of a certain tag, it first has to send a request to the tag in order to retrieve the ‘server URI’ in the localization relay object and learn the address of the relevant location resources at the backend server. After that, it can start retrieving or observing the position-related resources available at this server. The interaction model for this architecture is provided in Figure 5.

The second approach is based on a localization server that is exposing a single position object instance, which will be used for all of the tags; whereas, on the tag side, the location relay object with both the ‘server URI’ and ‘tag ID’ resources is used. The server URI holds a link to the position object instance in the localization server. Therefore, this model creates a an N-to-one link between tags and the localization server. Any client or application that reads the server URI and tag ID can interact with the localization server and retrieves the position data of the tag. In the case of a request on the position instance, the localization server will return the latest position update of whichever the positioned tag is. This enables very efficient operations when an application would like to track all of the tags. In this case, the tag ID can be used in order to match position information with the corresponding tag.

Using the third approach, the support for several localization servers is considered. In this case, the backend server exposes several (k) position object instances, while each target only needs to expose a location relay object containing the ‘tag ID’ resource. This approach creates a loosely-coupled architecture, where the ‘tag ID’ resource available at the tag is the only information available in order to match the tag and the position data obtained from different servers. With this approach, an application that is interested in tag locations can discover all tracked tags and find out their ‘tag ID’, as well as additional information exposed via other LwM2M resources. In a similar way, it can also discover all localization servers, as these behave as LwM2M devices, as well. Next, it can start retrieving notifications for a tag from several localization servers. Then, by combining the discovered tag information with the location update data, the application has all required information for further processing and taking actions.

Lastly, if one would like to make a localization server or localized tags, with a proprietary API, discoverable for interested third parties, then localization relay and localization server object (3362) can be used. The localized tags will expose a localization relay object instance, which is linked to a localization server object instance at the backend server. Within this localization server object instance, the exposed localization server resource will be used to describe the proprietary server API in JSON format. Any client application can discover these localization servers and tags, learn the used API and start retrieving the position data with a certain level of integration cost.

Although these four approaches use the same object, the relay object, at the tag, they differ in the sense of the resources they expose. Therefore, a client that discovers the resources at the tag can understand which model is used. If the tag exposes a server URI resource, linked to a position instance, but not tag ID, then it is based on the one-to-one approach. In case it exposes tag ID, but not server URI, then the loosely-coupled approach is followed. Thirdly, if it exposes both resources, this means the N-to-one approach is implemented by the localization system. Finally, if the tag exposes server URI linked to a localization server instance, then it is based on a proprietary API. Optionally, the first three approaches can also expose the localization object instance in order to advertise their existence or more information about the localization system by means of technology-related resources (5508 to 5515, 5552, 5701 etc.). The objects and necessary resources used for each approach are presented in Table 6. In this table, the number after ’*’ represents the number of instances for the given object that needs to be exposed by the corresponding device. In addition, N is the total number of the Tags, while k represents only a portion of these Tags.

## 6. Case Study: Hybrid Connected Warehouses

In the HyCoWareproject [24], we target the interconnection of heterogeneous systems in warehouses, encompassing systems of multiple vendors, aiming to create IoT readiness for industrial warehouses. For that purpose, we aim to make use of open IoT technologies to ease the deployment and interconnection between different solutions for connected goods, transport systems and operators. Since the targeted use cases require location information in order to further automate the warehouse operations or increase the visibility of certain objects, localization technologies and their integration into upcoming IoT solutions are key in the HyCoWare project.

- Hybrid tag: The first target system encompasses the design of a hybrid tag, which will be used to improve the visibility of industrial trolleys. During warehouse operations, the transport trolleys, with tags attached, will be monitored (position, temperature, humidity, etc.) by a control unit, which is closely interconnected with other systems. For this purpose, the tag needs to be equipped with wireless communication and indoor/outdoor localization technologies.  
- Connected operator: Another target product is the connected operator, which consists of an operator interface and navigation system to enable an operator to dynamically monitor and interact with other connected products. The location of the operators will be also tracked in order to navigate and direct them to the most relevant operations. Ideally, commercially-available PDAs or smart glasses are used as operator devices. Therefore, the operator needs to be located using technologies that are already part of these products, such as GPS, WiFi or BLE.  
- Connected conveyor: The last system is the chain conveyor system, which enables a finer tracking of pallets, carts and roll containers within the warehouse, as well as active involvement of the connected operators in the material flow. For this purpose, the transported pallets and carts have to be tracked by a localization system in addition to already existing RFID technology.

**Figure 6:**
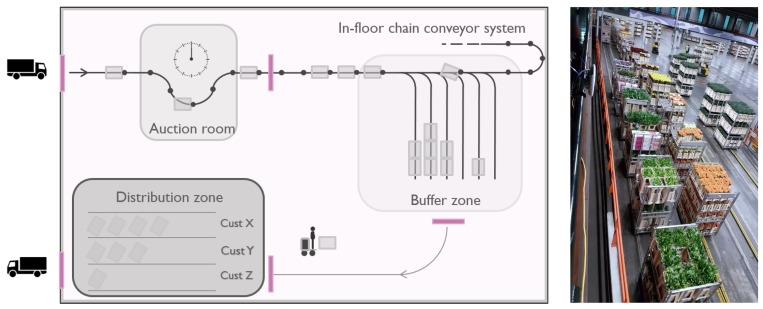
Sample warehouse floor plan with various zones.

Generally, warehouses consist of various zones or areas with different purposes, features or characteristics that mandate different requirements, such as location accuracy, update rate and many others [25]. For instance, at warehouse gates or docks, local presence detection is sufficient for incoming or outgoing goods, whereas accurate localization is needed inside critical handling and storage zones. On the other hand, coarse localization can be used to track goods outdoors or detect on or off premises. This diversity of warehouse sections is represented with a sample floor plan in Figure 6, which includes various zones with different accuracy requirements (illustrated with color density) and the gates between these zones and at the entrance and the exit.

### 6.1. Overall System Architecture

**Figure 7:**
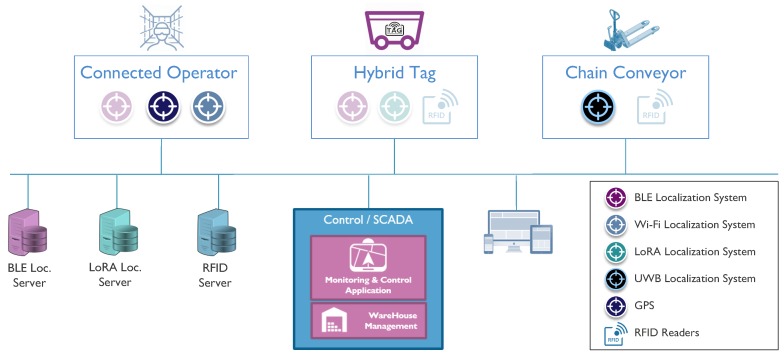
System architecture for the target use case.

Taking the application requirements and the characteristics of warehouses as the input, we came up with the system design and architecture, with various localization technologies, given in Figure 7. For the connected operator case, the targeted location technologies are BLE, WiFi and GPS, as these technologies are already available in many commercial PDAs. While GPS is going to provide outdoor location, BLE- and WiFi-based localization technologies will be used in order to obtain the indoor location with different accuracies. For the hybrid tag, the combination of BLE (optionally UWB), LoRa and RFID localization technologies will be used in order to track trolleys inside and outside venues of the target warehouse. For the chain conveyor system, UWB-based localization technology, jointly with RFID, will be used to track carts and pallets moving around the warehouse.

### 6.2. Modeling as LwM2M Devices

Thanks to powerful LwM2M management functionalities, LwM2M-compliant applications will be able to access not only position data, but also other device- (e.g., security, connectivity) and application- (e.g., temperature, humidity) related resources exposed on the same interface. However, due to the focus of this paper, we only provide the utilized location-related LwM2M models for the target devices described in the previous section, in Table 7.

As is presented in this table, the connected operator devices expose a single instance of the position object for WiFi-based localization, an IPSO GPS location object for GPS data and finally a location relay object for BLE-based localization technology in order to expose a web link to the localization server, which holds a position object for the operator. While hybrid tags have three instances of the localization relay object for all localization technologies (BLE, LoRa, RFID), it holds. Finally, the connected conveyors expose a position object instance and a localization relay object instance for UWB and RFID technologies, respectively.

On the other side, as the location is calculated by a backend server for LoRa, BLE- and RFID-based localization technologies, the backend server of each technology exposes necessary location object instances. For LoRa- and RFID-based technologies, we applied the infrastructure backend loosely-coupled interaction model; therefore, they expose several instances of the position object; while for BLE, the localization server uses the one-to-one interaction model and again exposes multiple position object instances for tracked devices.

### 6.3. Implementation and System Realization

**Figure 8:**
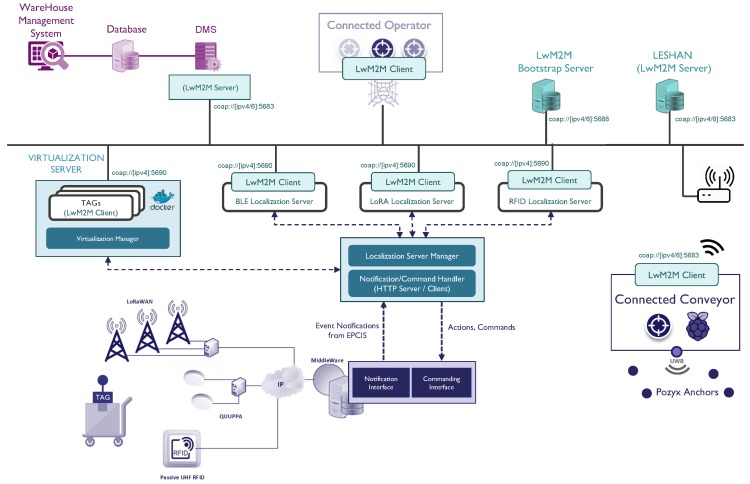
The system implementation for the target use case.

In order to demonstrate the targeted interoperability functionalities and realize the integrated ecosystem defined in the case study, we prepared a demonstration and validation setup along with the prototypes of the target products with localization capabilities. The resulting system architecture and design are provided in Figure 8.

For the implementation of the LwM2M client, we used an open source standard-compliant implementation, Anjay [26], that implements the LwM2M APIs for bootstrapping, registration, etc., as well as several standardized data models, and we extended this implementation with custom object models (defined in this paper) related to location data. For the LwM2M and bootstrap server implementation, we used another open source server implementation in Java, Leshan [27], and extended it again with the mentioned location-related objects.

Initially, the Warehouse Management System (WMS) and Device Management System (DMS) (for more information, refer to [28]) constitute the beating heart of the integrated warehouse ecosystem. This proprietary management system is able to discover and interact with all of the LwM2M-compliant devices by means of an embedded LwM2M server and eventually retrieve location or other application data. By combining location information with management capabilities, the WMS and DMS allows users to track and visualize actors and states, create tasks based on events and schedule them based on the state and location of actors, send commands and receive feedback from operators, calculate and visualize pathfinding data and navigate the actors accordingly.

**Figure 9:**
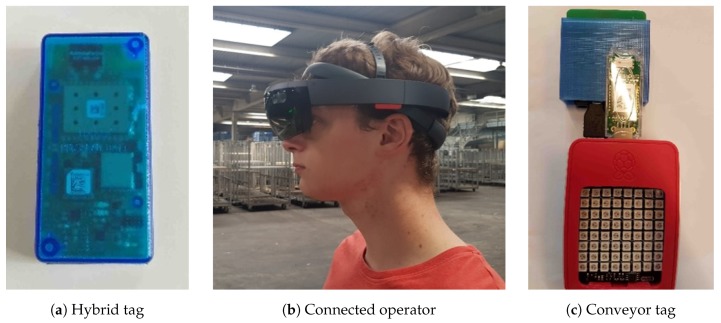
Prototypes for target use cases.

Regarding other members of this ecosystem, we used a newly-developed hybrid tag prototype, illustrated in Figure 9a (for more information, refer to [29]), in order to monitor the transport trolleys, with tag attached, during warehouse operations. As is illustrated in Figure 7, to overcome the integration of these hybrid tags, which cannot support LwM2M connectivity, we introduced the idea of device virtualization where we create LwM2M-compliant APIs. These virtualized devices are realized as Docker containers, which can be easily deployed on any operation system. Inside these containers, there are Anjay LwM2M clients running and exposing device- and location-related resources, which are received from a proprietary notification server and exposed to the LwM2M world. Similarly, for the localization servers (infrastructure backend localization systems), we deployed Lwm2M clients for each technology, which also receive the location data from the same notification server and expose it as LwM2M resources (3360). For the connected operator, an operator interface is developed by means of a tablet and, alternatively, virtual reality glasses, as presented in Figure 9b (for more information, refer to [28]), which enables an operator to dynamically monitor and react to tasks, monitor and interact with other connected products. Finally, for connected conveyors, we developed a prototype, presented in Figure 9c, which can be used to track pallets and carts in an accurate way, by means of an attached Pozyx tag, and provide virtual commands and messages via an attached LED matrix. Within this integrated ecosystem, all of the applications (location, light, text display, etc.) and management (device, server, etc.) traffic is modeled and realized by means of LwM2M interconnectivity.

**Figure 10:**
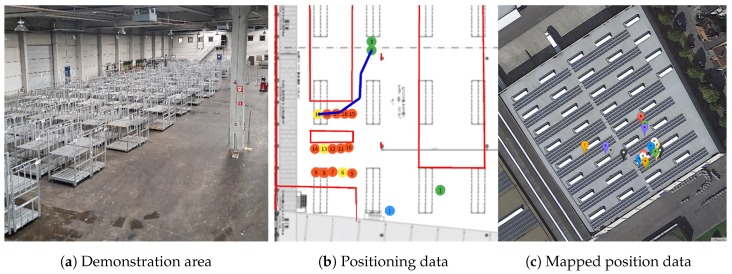
The demonstration of light-weight integration of localization systems targeting the hybrid connected warehouses.

The designed interconnected system and products are installed in a real flower auction warehouse, namely Euroveiling Flower Auction Center in Brussels, Belgium, and we demonstrated the interoperation functionalities with real warehouse scenarios, which include various zones and areas with different purposes, features or characteristics that mandate different localization requirements. The demonstration area is provided in Figure 10a, and two screenshots from location-aware warehouse operations are provided in Figure 10b,c, which includes position data from different technologies and devices. In Figure 10b, green circles represent the operators, blue shows the connected conveyor cart, red ones are transport trolleys and yellow circles are again transport trolleys, which require an action from the operators. Furthermore, the blue line is the calculated route for Operator 2 to navigate him to the next task (Trolley 19). Finally, Figure 10c represents the translated/mapped position data to GPS coordinates based on the reference point resources exposed by each localization technology on the top demonstration building.

### 6.4. System Validation

In order to validate the interoperation of localization technologies in the context of IoT, we present three use cases from the HycoWaRe project where we illustrate how the targeted functionalities can be achieved via the LwM2M interoperability model that we have defined.

#### 6.4.1. Use Case I: Fine Tracking for Trolleys

In this use case, we face a scenario where mobile trolleys must be tracked inside the warehouse by means of attached hybrid tags and monitored by a control and monitoring application. As is described in the previous section, the hybrid tag supports three different localization technologies with different localization capabilities. Initially, RFID readers are used to detect the arrival or departure of the trolleys for a certain zone in the warehouse. LoRa-based localization is used for non-critical areas, which require relatively low localization accuracy and update rate. Finally, the BLE-based localization technology is only deployed inside the buffer zone, where sub-meter accuracy is required and achieved by means of angle-of-arrival BLE localization.

We use the interaction and data models provided in the previous section for the hybrid tag. After commissioning a hybrid tag, the control application initially discovers the localization relay objects for each localization technology and the localization servers that expose the location data. After this discovery process, the application sends an observe request for the corresponding location resources and starts receiving position updates for all of the tags attached to the trolleys.

**Figure 11:**
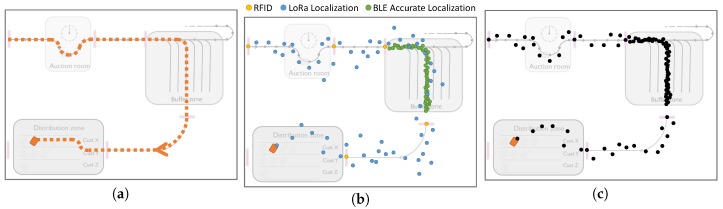
Use Case I: Fine tracking for trolleys. (**a**) Real track of the moving trolley; (**b**) Location measurements from three technologies; (**c**) Measured track after filtering.

We considered a scenario where a trolley is entering the warehouse and moves across the auction room, buffer zone and finally the distribution zone, with the resulting trajectory shown in Figure 11a. During this process, the trolley is being localized by all localization technologies, and these localization measurements are provided in Figure 11b. As this figure presents, the BLE-based localization measurements are only available in the buffer zone, while LoRa measurements are obtained for the whole warehouse, including the buffer zone. In the buffer zone, where trolleys are tracked by more than one localization technology, the cooperation of multiple localization technologies can improve location accuracy. In such a scenario, these localization technologies can cooperate and exchange data in order to improve location accuracy, overcome temporary failures or omit incorrect location data. Algorithm 1 roughly provides a simple algorithm, which can be used to combine the measurements of various localization technologies. The filtered and post-processed location data are provided in Figure 11c.

**Algorithm 1** Localization calculation for a device with multiple localization technology.
 1:*For every position update, do*: 2:
newPos[X,Y]←[XValue,YValue]
 3:
accuracy←[Uncertainty]
 4:
timestamp←[Timestamp]
 5:**if**currentPosition=null**then**                    ▹ First position measurement 6: currentPosition←newPos[X,Y] 7: lastPositionUpdate←timestamp 8: **return** currentPosition 9:
**end if**
10:**if**timestamp−lastPositionUpdate>maxAge**then**  ▹ No other measurements for post-processing11: currentPosition←newPos[X,Y]12: lastPositionUpdate←timestamp13:
**else**
14: currentPosition←calculateNewPosition(newPos[X,Y],timestamp,accuracy) ▹ Post-processing or filtering15: lastPositionUpdate←timestamp16:
**end if**
17:
**return** currentPosition



Another useful feature in this scenario is that different localization technologies can activate or deactivate each other in order to save resources. For instance, an RFID reader at the gate of the buffer zone or the LoRa-based low power localization technology detects an object that starts moving in the buffer zone, subsequently enabling a more accurate localization system (BLE in our scenario) for more precise tracking.

#### 6.4.2. Use Case II: Position Translation and Mapping

**Figure 12:**
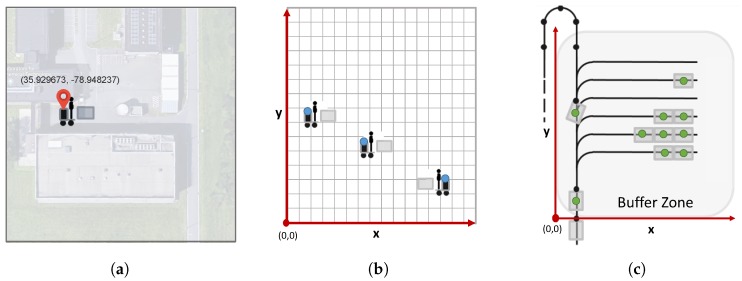
Different localization technologies with different coordinate systems (**a**) GPS position of the operator; (**b**) Indoor Localization 1; (**c**) Indoor Localization 2.

The second use case is targeting the position translation and coordinate mapping across multiple technologies. For this use case, we consider a scenario where a connected operator would like to monitor the location of other operators, connected conveyor carts and trolleys in the warehouse. However, as is described above, all of these devices use a variety of indoor and outdoor localization technologies, which probably have different coordinate systems. Therefore, in order to realize the target scenario, there is a need for a mapping and translation process not only between non-spatial and spatial data, but also between different coordinate systems. For instance, Figure 12 presents the output for three independent localization technologies.

**Figure 13:**
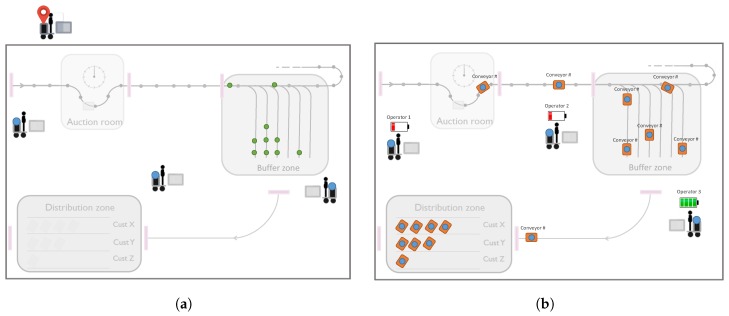
(**a**) The localization after translation and mapping; (**b**) Sample application for the combination of position data with LwM2M semantic information.

In this sense, we can define a reference coordinate system (or use one of the coordinate systems from any of the localization technologies) and translate all of the non-spatial or spatial location data into this coordinate system by using the provided resources in position object (latitude, longitude, altitude, compass direction, elevation direction) in order to specify the actual position of the reference point and the relative orientation of the measurement area with respect to this reference point. The pseudo algorithm for the coordinate translation between the two coordinate systems is provided in Algorithm 2. In this algorithm, initially, the great-circle distance based on the Haversine formula [30] and the bearing [31] between the two reference points are calculated, which are then translated into the x and y offset values. The compass direction difference between the two reference points is also calculated. After that, the mapped coordinate values are obtained by using the compass direction difference and offset coordinate values of the reference points. The outcome of a mapping operation for the three location data from Figure 12 is provided in Figure 13a.

**Algorithm 2** Location mapping/translation.
 1:$\lambda_{\mathrm{base}},\gamma_{\mathrm{base}},\theta_{\mathrm{base}}$              ▹ Latitude, longitude, compass direction of base coordinate system 2:*For every position update, do*: 3:
$\left[x_{\mathrm{new}},y_{\mathrm{new}}]\leftarrow [XValue,YValue\right]$
 4:**if**$\lambda_{\mathrm{ref}}=null$**then**                        ▹ First position update for the technology 5: $\left[\lambda_{\mathrm{ref}},\gamma_{\mathrm{ref}},\theta_{\mathrm{ref}},{altitude}_{\mathrm{ref}}]\leftarrow retrieveReferencePoint(\right)$ 6: $\mathrm{earthRadius}\leftarrow getRadius(\lambda_{\mathrm{ref}},{altitude}_{\mathrm{ref}})$                 ▹ [6356.752km : 6378.137km] 7: $\Delta \lambda \leftarrow \lambda_{\mathrm{ref}}*\pi /180-\lambda_{\mathrm{base}}*\pi /180$ 8: $\Delta \gamma \leftarrow \gamma_{\mathrm{ref}}*\pi /180-\gamma_{\mathrm{base}}*\pi /180$ 9: $\beta =\sin^{2}(\Delta \lambda /2)+\cos(\lambda_{\mathrm{base}}*\pi /180)*\cos(\lambda_{\mathrm{ref}}*\pi /180)*\sin(\delta \gamma /2)*\sin(\Delta \gamma /2)$10: $\epsilon =2*atan2(\sqrt[{2}]{\beta },\sqrt[{2}]{1-\beta })$11: distance←earthRadius∗ϵ∗100012: $\mathrm{bearing}\leftarrow atan2(\cos(\lambda_{\mathrm{base}})*\sin(\lambda_{\mathrm{ref}})-\sin(\lambda_{\mathrm{base}})*\cos(\lambda_{\mathrm{ref}})*\cos(\gamma_{\mathrm{ref}}-\gamma_{\mathrm{base}}),\sin(\gamma_{\mathrm{ref}}-\gamma_{\mathrm{base}})*\cos(\lambda_{\mathrm{ref}}))$13: $x_{\mathrm{offset}}\leftarrow distance*\sin(bearing))$14: $y_{\mathrm{offset}}\leftarrow distance*\cos(bearing))$15: $\Delta \theta \leftarrow \theta_{\mathrm{ref}}-\theta_{\mathrm{base}}$16:
**end if**
17:
$x_{\mathrm{mapped}}\leftarrow x_{\mathrm{new}}*\cos(\Delta \theta )-y_{\mathrm{new}}*\sin(\Delta \theta )+x_{\mathrm{offset}}$
18:
$y_{\mathrm{mapped}}\leftarrow x_{\mathrm{new}}*\sin(\Delta \theta )+y_{\mathrm{new}}*\cos(\Delta \theta )+y_{\mathrm{offset}}$
19:$plot(x_{\mathrm{mapped}},y_{\mathrm{mapped}})$                          ▹ Mapped/translated location data20:
**return**



#### 6.4.3. Use Case III: Combining with other LwM2M Semantic Information

The last use case is about the easy combination of position data with other application- and device-related semantic data. As mentioned before, the usage of LwM2M enables our applications and devices to access not only position data, but also other device- (e.g., security, connectivity, battery level) and application- (e.g., temperature, humidity) related resources exposed on the same interface. For instance, if a control application would like to know all of the operators in a certain zone, it can retrieve the device information and position information via LwM2M and filter only the operator devices within the target zone; or a monitoring unit can monitor the position, temperature and humidity of a transport trolley at the same time without any need for an extra interface. A sample preview of the interconnected data plane is provided in Figure 13b.

### 6.5. Discussion about the Case Study

As mentioned, the objective of this case study is to enable fully-automated and smart warehouses by means of the interconnection of these and any other heterogeneous systems of multiple vendors. For that purpose, we make use of open IoT technologies to ease the deployment of and interconnection between different solutions and multiple localization technologies, which are able to track thousands of objects and also interoperate seamlessly and spontaneously. The outcome of the case study shows that such an integration and interoperation will enable location-aware applications (in the context of IoT) to improve location accuracy, overcome temporary failures or omit incorrect location data, to translate several pieces of location information into a reference coordinate system and finally combine LwM2M semantic capabilities with location information without any extra effort.

## 7. Conclusions

Despite their significant potential in IoT applications, the amount of research targeting the integration of indoor localization technologies in real-life IoT applications is limited. In this work, we investigated the semantic interoperation and integration of different positioning systems in the context of IoT and validated our approach based on a real IoT case study.

In order to overcome the seamless and spontaneous interoperation of localization systems and their interconnection with minimized integration cost, we focused on open IoT technologies and defined interfaces for these products based on LwM2M/IPSO specifications. Our design is clean and able to support all interaction models we have encountered in today’s localization systems. In addition, it is able to handle a wide variety of connected actors, as illustrated by our use case, as well as localization technologies, despite any kind of variation in their nature. Therefore, we can conclude that IoT protocols already offer powerful and efficient mechanisms in order to realize all of these functionalities. We believe this work can provide a baseline, for the localization system developers, about how to use IoT protocols and platforms in order to integrate their products in IoT applications. It can also help IoT system providers to understand the characteristics and needs of different localization technologies in order to realize their semantic and structural interoperability in the context of IoT.

## Figures and Tables

**Table 1:** LwM2M/IPSO objects related to location data.

ID	Name	Instances	Mandatory	Submitter	Description
3336	GPS location	Multiple	Optional	IPSO	The GPS location object defined by the IPSO Alliance
3360	Position object	Multiple	Optional	Created	The proposed position object model for localization systems with coordinates and reference point resources
3361	Localization relay object	Single	Optional	Created	The proposed object model to expose all necessary resources to associate and link a localization server
3362	Localization server object	Single	Optional	Created	The proposed object model to be used to represent a localization server that tracks several objects

**Table 2:** IPSO location object for GPS data (Object ID 3336) [16].

ID	Name	Operations	Mandatory	Type	Submitter	Description
5514	Latitude	R	Mandatory	String	IPSO	The decimal notation of latitude, e.g., −43.5723
5515	Longitude	R	Mandatory	String	IPSO	The decimal notation of longitude, e.g., 153.21760
5516	Uncertainty	R	Optional	String	IPSO	The accuracy of the position
5705	Compass direction	R	Optional	Float	IPSO	Measured direction (0–360 deg.)
5705	Velocity	R	Optional	Opaque	IPSO	The velocity of the device
5518	Timestamp	R	Optional	Time	IPSO	The timestamp of the location measurement
5750	Application Type	R/W	Optional	String	IPSO	The application as a string

**Table 3:** Position object based on LwM2M/IPSO (Object ID 3360).

ID	Name	Operations	Mandatory	Type	Submitter	Description
5702	*X* Value	R/W	Mandatory	Float	IPSO-Updated	The measured value along the *X* axis.
5703	Y value	R/W	Mandatory	Float	IPSO-Updated	The measured value along the *Y* axis.
5704	*Z* value	R/W	Optional	Float	IPSO-Updated	The measured value along the *Z* axis.
5508	Min *X* value	R/W	Optional	Float	IPSO-Updated	The minimum value along the *X* axis
5509	Max *X* value	R/W	Optional	Float	IPSO-Updated	The maximum value along the *X* axis
5510	Min *Y* value	R/W	Optional	Float	IPSO-Updated	The minimum value along the *Y* axis
5511	Max *Y* value	R/W	Optional	Float	IPSO-Updated	The maximum value along the *Y* axis
5512	Min *Z* value	R/W	Optional	Float	IPSO-Updated	The minimum value along the *Z* axis
5513	Max *Z* value	R/W	Optional	Float	IPSO-Updated	The maximum value along the *Z* axis
5514	Latitude (Reference Point)	R/W	Optional	String	IPSO-Updated	The decimal notation of latitude, e.g., −43.5723
5515	Longitude (Reference Point)	R/W	Optional	String	IPSO-Updated	The decimal notation of longitude, e.g., 153.2176
5552	Altitude (Reference Point)	R/W	Optional	Float	Created	The altitude of the reference point
5705	Compass direction	R/W	Optional	Float	IPSO-Updated	The compass direction for the measurement area relative to reference point (0–360 deg)
5553	Elevation direction	R/W	Optional	Float	Created	The elevation direction for the 3D measurement area relative to the reference point
5516	Uncertainty	R/W	Optional	Float	IPSO-Updated	The accuracy of the position
5518	Timestamp	R/W	Optional	Time	IPSO-Updated	The timestamp of the location measurement
5701	Sensor Units	R/W	Optional	String	IPSO-Updated	Measurement units’ definition e.g., “meters”
5554	Server URI	R/W	Optional	String	Created	URI that identifies the localization server (weblinking)
5555	Target ID	R/W	Optional	Integer	Created	The unique ID assigned by localization system
5556	Update flag	R/W	Optional	Boolean	Created	The flag to be set upon a position update (write) by a localization server
5556	Update flag	R/W	Optional	Boolean	Created	The flag to be set upon a position update (write) by a localization server
5750	Application Type	R/W	Optional	String	IPSO	The application as a string

**Table 4:** Localization relay object based on LwM2M/IPSO (Object ID 3361).

ID	Name	Operations	Mandatory	Type	Submitter	Description
5554	Server URI	R	Mandatory	String	Created	URI that identifies the localization server (WebLinking)
5555	Target ID	R/W	Optional	Integer	Created	The unique ID assigned by the localization system
5750	Application type	R/W	Optional	String	IPSO	The application as a string, e.g., “UWB positioning”

**Table 5:** Localization server object based on LwM2M/IPSO (Object ID 3362).

ID	Name	Operations	Mandatory	Type	Submitter	Description
5557	Localization server	R	Mandatory	String	Created	The API description (in JSON format) of the proprietary localization server
5750	Application type	R/W	Optional	String	IPSO	The application as a string, e.g., “UWB positioning”

**Table 6:** LwM2M objects and resources exposed for each interaction model.

Interaction Model	Tag			Localization Server	
	Object ID	Server URI	Tag ID	Object ID	Tag ID
Self-Positioning	3360	**✗**	**✗**	-	-
Self-oriented Infrastructure-Assisted	3360	**✗**	**✓**	-	-
Infrastructure Backend One-to-One	3361	**✓**	**✗**	3360 ^*N^	**✓**
Infrastructure Backend N-to-One	3361	**✓**	**✓**	3360	**✓**
Infrastructure Backend Loosely Coupled	3361	**✗**	**✓**	3360 ^*k^	**✓**
Infrastructure Backend Proprietary API	3361	**✓**	**✗**	3362	-

**Table 7:** The object models for LwM2M devices in HyCoWaRe.

Device Name	Localization Technology	Interaction Model	Object Type
**Connected Operator**	BLE-based Localization Technology	Infrastructure Backend One-to-One	3361
	WiFi-based Localization Technology	Self-Positioning Technology	3360
	GPS	Self-Positioning Technology	3336
**Hybrid Tag**	BLE-based Localization Technology	Infrastructure Backend One-to-One	3361
	LoRa-based Localization Technology	Infrastructure Backend Loosely Coupled	3361
	RFID	Infrastructure Backend Loosely Coupled	3361
**Connected Conveyor**	UWB Localization Technology	Self-Positioning Technology	3360
	RFID	Infrastructure Backend Loosely Coupled	3361
**BLE Localization Server**	BLE Localization Technology	Infrastructure Backend One-to-One	3360
**RFID Localization Server**	RFID Localization Technology	Infrastructure Backend Loosely Coupled	3360
**LoRa Localization Server**	LoRa Localization Technology	Infrastructure Backend Loosely Coupled	3360

## References

[1] Li S., Xu L.D., Zhao S. (2015). The Internet of Things: A Survey. Information Systems Frontiers.

[2] Al-Fuqaha A., Guizani M., Mohammadi M., Aledhari M., Ayyash M. (2015). Internet of Things: A Survey on Enabling Technologies, Protocols, and Applications. IEEE Commun. Surv. Tutor..

[3] Dardari D., Closas P., Djurić P.M. (2015). Indoor Tracking: Theory, Methods, and Technologies. IEEE Trans. Veh. Technol..

[4] Alarifi A., Al-Salman A., Alsaleh M., Alnafessah A., Al-Hadhrami S., Al-Ammar M.A., Al-Khalifa H.S. (2016). Ultra Wideband Indoor Positioning Technologies: Analysis and Recent Advances. Sensors.

[5] Liu H., Darabi H., Banerjee P., Liu J. (2007). Survey of Wireless Indoor Positioning Techniques and Systems. IEEE Trans. Syst. Man Cybern. Part C Appl. Rev..

[6] Semtech Corporation (2016). LoRa Geolocation Solution for Low Power Wide Area Networks. http://www.semtech.com.

[7] OneM2M (2015). White Paper: The Interoperability Enabler for the Entire M2M and IoT Ecosystem. http://www.onem2m.org/images/files/onem2m-whitepaper-january-2015.pdf.

[8] Open Mobile Alliance. http://openmobilealliance.org.

[9] IPSO Alliance, Internet Protocol for Smart Objects (IPSO). https://www.ipso-alliance.org.

[10] Open Mobile Alliance (2017). Lightweight Machine to Machine Technical Specification, Approved Version 1.0.

[11] Wang X., Shang J., Yu F., Yan J. (2013). Indoor Semantic Location Models for Location-Based Services. Int. J. Smart Home.

[12] Hu H., Lee D.-L. Semantic Location Modeling for Location Navigation in Mobile Environment. Proceedings of the IEEE International Conference on Mobile Data Management.

[13] Roth J. (2003). Flexible positioning for location-based services. IADIS Int. J. WWW/Internet.

[14] Veeckman C., Jedlička K., De Paepe D., Kozhukh D., Kafka Š., Colpaert P., Čerba O. (2017). Geodata interoperability and harmonization in transport: A case study of open transport net. Open Geospat. Data Softw. Stand..

[15] Shelby Z., Hartke K., Bormann C. (2014). The Constrained Application Protocol (CoAP). Internet-Draft Draft-Ietf-Core-Coap-18.

[16] OMNA Lightweight M2M (LwM2M) Object & Resource Registry. http://www.openmobilealliance.org.

[17] Jimenez J., Koster K., Tschofenig H. (2016). IPSO Smart Objects.

[18] Zhang X., Bae H.Y. (2015). Location Positioning and Privacy Preservation Methods in Location-based Service. Int. J. Secur. Its Appl..

[19] Van der Stok P., Bormann C., Sehgal A. (2017). Patch and Fetch Methods for the Constrained Application Protocol (CoAP). Internet-Draft Draft-Ietf-Core-Etch-04.

[20] (2018). Open Mobile Allience LwM2M v1.1 New Features Preview. http://openmobilealliance.org/iot/lightweight-m2m-lwm2m/lightweightm2m-1-1-preview-3.

[21] Hartke K. (2014). Observing Resources in the Constrained Application Protocol (CoAP). Internet-Draft Draft-Ietf-Core-Observe-16.

[22] Pozyx Accurate Positioning. https://www.pozyx.io.

[23] Quuppa Intelligent Locating System. http://quuppa.com.

[24] Hycoware Project, 2016–2018. https://www.imec-int.com/nl/imec-icon/research-portfolio/hycoware.

[25] Karaagac A., Haxhibeqiri J., Ridolfi M., Joseph W., Moerman I., Hoebeke J. Evaluation of Accurate Indoor Localization Systems in Industrial Environments. Proceedings of the 22nd IEEE International Conference on Emerging Technologies And Factory Automation.

[26] Anjay: Open-source LwM2M Library. https://www.avsystem.com/products/anjay.

[27] Eclipse Leshan: An OMA Lightweight M2M (LWM2M) Implementation in Java. https://eclipse.org/leshan.

[28] Intation: Innovates Innovation. https://www.intation.eu.

[29] Aucxis CVBA. https://www.aucxis.com.

[30] Robusto C.C. (1957). The cosine-haversine formula. Am. Math. Mon..

[31] Veness C. (2012). Movable Type Scripts: Calculate Distance, Bearing and More Between Latitude/longitude Points. https://www.movable-type.co.uk/scripts/latlong.html.

